# Numerical performance of CO_2_ accumulation and droplet dispersion from a cough inside a hospital lift under different ventilation strategies

**DOI:** 10.1038/s41598-024-57425-z

**Published:** 2024-03-21

**Authors:** Sergio A. Chillón, Unai Fernandez-Gamiz, Ekaitz Zulueta, Ainara Ugarte-Anero, Jesus Maria Blanco

**Affiliations:** 1 Energy Engineering Department, School of Engineering of Vitoria-Gasteiz, University of the Basque Country, UPV/EHU, Nieves Cano 12, 01006 Vitoria-Gasteiz, Araba Spain; 2 Automatic and Simulation Department, University of the Basque Country, UPV/EHU, Nieves Cano 12, 01006 Vitoria-Gasteiz, Araba Spain; 3 Energy Engineering Department, School of Engineering, University of the Basque Country (UPV/EHU), Plaza Ingeniero Torres Quevedo, Building 1, 48013 Bilbao, Spain

**Keywords:** CFD, COVID-19, Interior ventilation, Droplet contagious, Airborne transmission, Cough, Hospital lift, CO_2_ transport, Environmental social sciences, Mechanical engineering

## Abstract

The impact of mechanical ventilation on airborne diseases is not completely known. The recent pandemic of COVID-19 clearly showed that additional investigations are necessary. The use of computational tools is an advantage that needs to be included in the study of designing safe places. The current study focused on a hospital lift where two subjects were included: a healthy passenger and an infected one. The elevator was modelled with a fan placed on the middle of the ceiling and racks for supplying air at the bottom of the lateral wall. Three ventilation strategies were evaluated: a without ventilation case, an upwards-blowing exhausting fan case and a downwards-blowing fan case. Five seconds after the elevator journey began, the infected person coughed. For the risk assessment, the CO_2_ concentration, droplet removal performance and dispersion were examined and compared among the three cases. The results revealed some discrepancies in the selection of an optimal ventilation strategy. Depending on the evaluated parameter, downward-ventilation fan or no ventilation strategy could be the most appropriate approach.

## Introduction

Several breathing pandemics have occurred in recent decades^1^. Moreover, there is a great likelihood of overcoming new pandemics in a few years^2^. The main routes for transmitting breathing diseases include direct transmission via exhaled droplets, fomite transmission through contact with contaminated surfaces, inhalation of active viral or bacterial particles and contact with nonvolatile residual nuclei^3–7^. Droplets and residual transmission are the main issues of several studies due to the complexity of preventing their transmission. The path along which a droplet travels depends on several factors, such as its initial velocity, the size of the particle, and other environmental parameters, such as relative humidity (RH), environmental temperature (T_∞_), and above all, the surrounding air velocity^8–14^. To further complicate the calculations, evaporation also plays an important role^15^. According to RH and T_∞_, the evaporation ratio can high or low, leading to residual salt particles floating in the local volume; this residual accumulation is known as aerosol, and according to several studies, this path can be assumed to be similar to a gas^16–20^. Thus, several studies have experimentally tracked human-emitted CO_2_ gas as an easy and inexpensive way to monitor aerosol dispersions in relation to the droplet solid residuals^21–26^.

Among the different origins of exhaled droplets, the most common and studied are speaking, coughing and sneezing^27–35^. A more violent exhalation correlated to more numerous and larger droplets; thus, the quantity of virus inside the droplet is presumed to increase by several orders of magnitude^36,37^. Understanding the physics of droplets of different sizes is vital for predicting droplet behaviour inside a certain volume. In those terms, Wells^38^ experimentally studied the evaporation of a single droplet in a freefall. Xie et al.^39^ expanded on this research and considered the effect of RH.

The application of computational fluid dynamics (CFD) simulations can affordability and realistically predict different risky scenarios and increase the understanding of the physics of droplets. Since the appearance of COVID-19, a large number of scientists have investigated the generation and dispersion of droplets and nuclei by using CFD; for example, Anzai et al.^36^ modelled the entire upper respiratory tract covered by a mucous film to recreate droplet formation during a cough, and Bahramian et al.^9^ recently marked the effect of T_∞_ on the velocity of droplets generated by a sneeze. Furthermore, a variety of health-related dangerous places were staged in different indoor environments with specific ventilation strategies, occupations, environmental conditions and breathing activities^14,40–47^. Other authors included public transport^10,12,48–50^, hospital environments^8,51,52^ and, more specifically, elevators^53–56^.

In the present study, a small hospital elevator was modelled via CFD to recreate the dispersion of cough droplets and the accumulation of CO_2_ caused by breathing during a journey of 15 s. The ambient conditions were 25 °C and 50% RH. Two standing people were introduced facing each other and 20 cm away from the longitudinal axis. Breathing was modelled for both occupants, and one was an asymptomatic infected person, who coughed at second 5 from the start. The lift was equipped with a ceiling fan. Three simulations were carried out (1) without ventilation (NF), (2) with upwards blowing ventilation (UF), and (3) with downwards blowing ventilation (DF).

The major novelty of this research is the quantitative approach of removing droplets and residual nuclei among the three ventilation setups inside a small clinical elevator. The numerical use of CO_2_ as an aerosol tracer has been limitedly used but was used for this study for computational cost savings while maintaining reliability and realistic results.

## Numerical method

### Case description

The current study consists of CFD simulations with a hospital lift as a scenario. The lift dimensions were the same as those previously used in^57^ and^58^; this type of elevator is 2.1 meters (m) long, 1.2 m wide and 2 m tall. These measurements are in accordance with the regulations of European Standards (EN) 81-20 and EN 81-50. The lift was modelled with a 30 cm diameter circular hole in the centre of the ceiling. This hole was modelled as a wall, an upwards blowing fan and a downwards blowing air inlet, according to the studied case. Shinohara et al.^50^ noted that the stream produced by a fan could be simplified by a simple hole. The air supply velocity through the fan was 6.5 meters/second (m/s) for both cases where ventilation was used. On one sidewall, three air supply racks were modelled; each rack was 0.01 centrimetre (cm) thick with a length of 1.5 m located 15 cm from the floor, and the vertical distance between each rack was 15 cm. The ambient inside was modelled under clinical conditions: 25 °C of temperature and 50% RH according to the UNE 100713:2005 normative.

**Figure 1:**
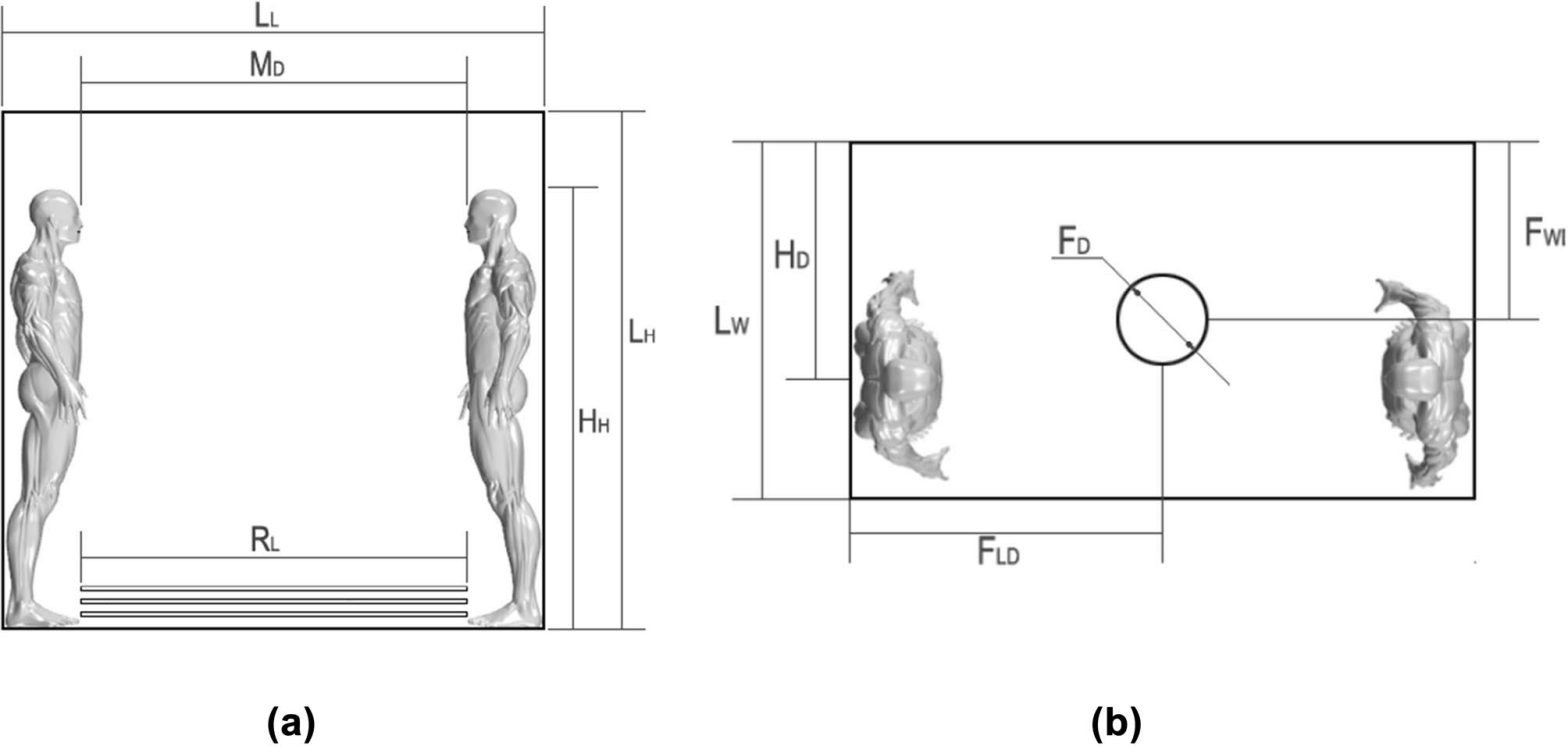
Dimensions of the computational domain: (**a**) Front view; (**b**) plan.

**Figure 2:**
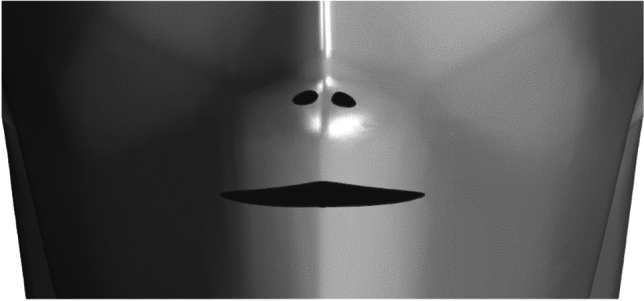
Details of the mouth and nose inlets.

Inside the lift, two 1.7 m tall humans were modelled face to face, spaced 1.5 m from each other. To reproduce breathing and coughing, human mannequins were configured with nose and mouth openings. The nostril outlets were two holes of elliptical appearance, approximately 1.7 cm in length and 0.5 cm in width. The mouth was a 4 cm long and 0.5 cm opening hole, in agreement with measurements from Dbouk and Drikakis^59^. More details are provided in Figs. 1, 2 and Table 1.

**Table 1:** Computational domain measurements.

Dimension	Meaning	Value
L_L_	Lift length	2.1 m
L_H_	Lift height	2 m
L_W_	Lift width	1.2 m
H_H_	Human height	1.7 m
M_D_	Mouth distance	1.5 m
H_D_	Human displacement	0.8 m
F_D_	Fan diameter	0.3 m
R_L_	Rack length	1.5 m
F_LD_	Fan length displacement	1.05 m
F_WD_	Fan width displacement	0.6 m

**Figure 3:**
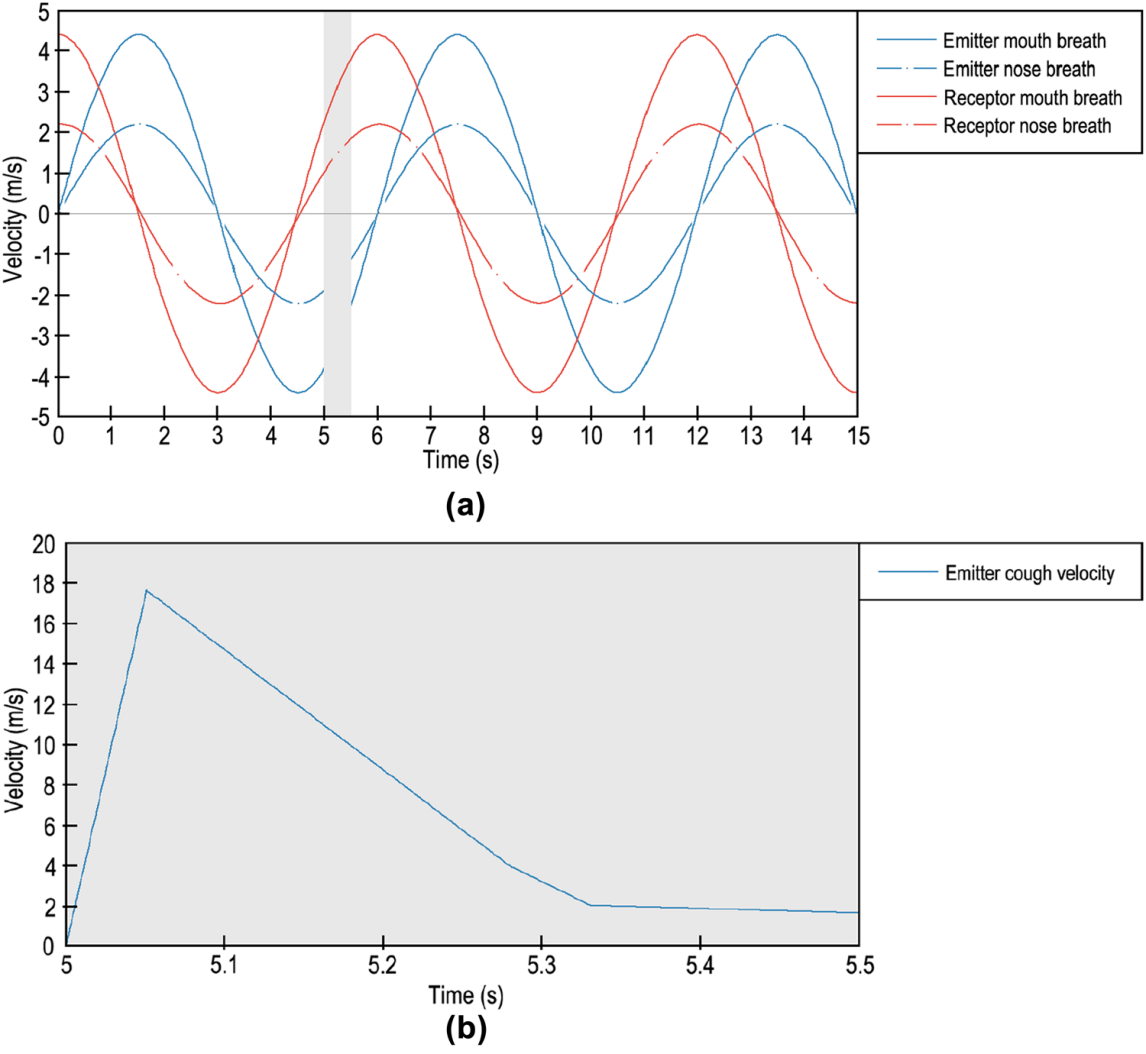
Inlet velocity parameters: (**a**) Breathing; (**b**) Coughing.

Figure 3a shows the velocity profiles of breathing and coughing with respect to the nose and mouth. The emitter breathing velocity profile was modelled as sinusoidal for exhalation and inhalation, with maximum peaks of 4.4 m/s for mouth breath and 2.2 m/s for nose breath, in accordance with Du and Chen^53^ and Mhetre et al.^60^. The breath was noncontinuous because the cough started at second 5 and ended at second 5.5. Similarly, the receptor breath was a cosine with the same velocity peaks. In this case, the breath was completely continuous at all times. The total cycle time of both breathings was 6 s. The pulmonary ventilation rate was 40.7 L/minute (33.6 L/minute across the mouth and 7.1 L/minute across the nose); this represents a ventilation rate that is near hyperventilation. The exhaled gases from the mouth were 78% N_2_, 17% O_2_ and 5% CO_2_, as noted in Hibbard et al.^61^. Only the cough emitter exhaled CO_2_, and this was modelled as the Eulerian phase, as done in different studies like in Pham et al.^62^ or Sadeghizadeh et al.^63^. The exhaled CO_2_ was only monitored from the cough emitter to observe the CO_2_ increase caused by only the infected person. The cough total volume was 0.64 L.

The cough velocity profile can be divided into four phases, as shown in Fig. 3b. In phase one, the air velocity immediately increased up to 17.7 m/s, and phase 2 showed a quick decrease lasting 0.23 s with an ending velocity of 3.9 m/s. Phase 3 showed a short decrease lasting 0.05 s with an ending velocity of 1.995 m/s. Phase 4 showed a slight decrease lasting 0.19 s with an ending velocity of 1.575 m/s. The total cough time was 0.5 s. This velocity profile was based on the study of Mahajan et al.^33^ and validated in^57^ and^58^.

### Meshing

**Figure 4:**
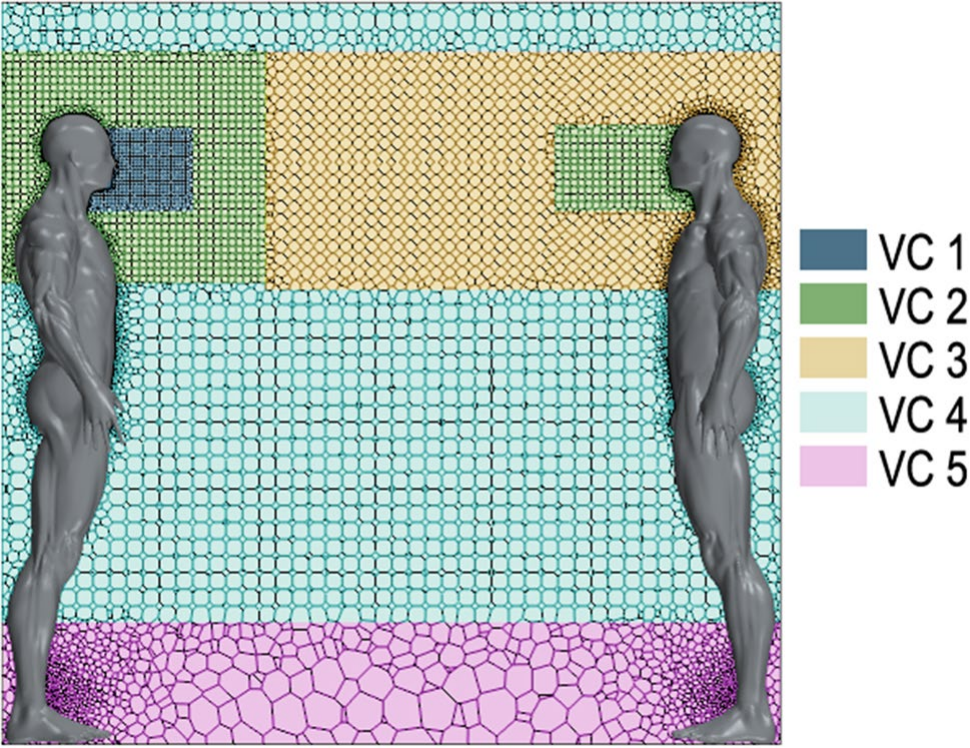
Mesh grid.

Due to the complex human body shapes, the computational volume was discretized using a polyhedral mesh of 667,917 cells. This mesh type is effectively coupled to different boundaries in a well-structured disposition. The mesh was divided into five volume controls (VC) with 5 different coarse levels. In Fig. 4, a transverse mesh plane is observed, along with the volume controls. The finest volume (VC1, 12,553 cells) was placed from the middle of the emitter’s head to 30 cm downstream in a 0.4 × 0.2 cm plane. VC2 (131,805 cells) was located in two areas (around VC1 and opposite from VC1), and the area opposite VC1 had the same volume and shape of VC1 but was located at the head of the receptor mannequin. VC3 (179,006 cells) was positioned between two humans. VC4 (311,497 cells) was located 15 cm from the ceiling and between 0.35 and 1.25 m from the floor. Finally, VC5 (33,056 cells), the coarsest grid, was placed at the bottom. A surface remesher was modelled for the two human bodies to obtain more accurate results for the mannequin shapes and to maintain an optimum geometry level.

To ensure the mesh-related independence of the numerical results, a mesh dependency study was performed using Richardson’s extrapolation, as explained in Roache^64^. For this purpose, three meshes (fine, medium and coarse) with different refinement levels were configured. The cell size ratio was r ≈ 2. In the study, the axial velocity was measured 10 cm downstream from the mouth of the emitter. Tables 2, 3, 4 provide the different results obtained for the case of 17.7 m/s (the maximum cough velocity) in steady convergence.

**Table 2:** Axial velocity results for each mesh.

Mesh	Number of cells	V_ax_ (m/s)
Fine	667,917	0.8700
Medium	268,331	0.8040
Coarse	149,527	0.2730

**Table 3:** Results for extrapolation and convergence order.

Parameter	Value
${\left({V}_{ax}\right)}_{h=0}$	0.8794
$p$	3.01

**Table 4:** Relative errors and CGI values of the proposed meshes.

Parameter	Value
${\epsilon }_{12}$	0.0758621
${\epsilon }_{23}$	0.6604478
${GCI}_{12}$	0.0134593
${GCI}_{23}$	0.1171762
$\frac{{GCI}_{23}}{{r}^{p}\cdot {GCI}_{12}}$	1,082,089,552

Equation (1) shows the calculation for the extrapolated velocity; ${\text{h}}=0$ represents the extrapolated most accurate result. Equation (2) was used to calculate the order of convergence for a three-mesh grid convergence index study. The obtained results can be found in Table 3.

1 $${\left({V}_{ax}\right)}_{h=0}={\left({V}_{ax}\right)}_{1}+\frac{{\left({V}_{ax}\right)}_{1}-{\left({V}_{ax}\right)}_{2}}{{r}^{p}-1}$$

2 $$p=\frac{{\text{ln}}\left(\frac{{\left({V}_{ax}\right)}_{3}-{\left({V}_{ax}\right)}_{2}}{{\left({V}_{ax}\right)}_{2}-{\left({V}_{ax}\right)}_{1}}\right)}{{\text{ln}}2}$$

where ${V}_{ax}$ is the axial velocity (m/s), $r$ is the cells incrementing ratio and $p$ is the order of convergence.

Equations (3) and (4) can be used to calculate the mesh relative error. Using the recommended value of FS = 1.25 for a three-mesh comparison, both fine and coarse GCIs are solved with Eqs. (5) and (6).

3 $${\epsilon }_{12}=\frac{{\left({V}_{ax}\right)}_{1}-{\left({V}_{ax}\right)}_{2}}{{\left({V}_{ax}\right)}_{1}}$$

4 $${\epsilon }_{23}=\frac{{\left({V}_{ax}\right)}_{2}-{\left({V}_{ax}\right)}_{3}}{{\left({V}_{ax}\right)}_{2}}$$

5 $${GCI}_{12}=\frac{FS\left|{\epsilon }_{12}\right|}{{r}^{p}-1}\cdot 100$$

6 $${GCI}_{23}=\frac{FS\left|{\epsilon }_{23}\right|{r}^{p}}{{r}^{p}-1}\cdot 100$$

where $\in$ is the relative error between two given results, $FS$ is the security factor and $GCI$ term is the grid convergence index result.

To check that our solution is within the asymptotic range, Eq. (7) is recommended. All the solutions can be found in Table 4.

7 $$\frac{{GCI}_{23}}{{r}^{p}\cdot {GCI}_{12}}\approx 1$$

Equation (7) shows that the fine mesh is reliable, with an estimated error of 1.346%.

### Numerical methods

**Figure 5:**
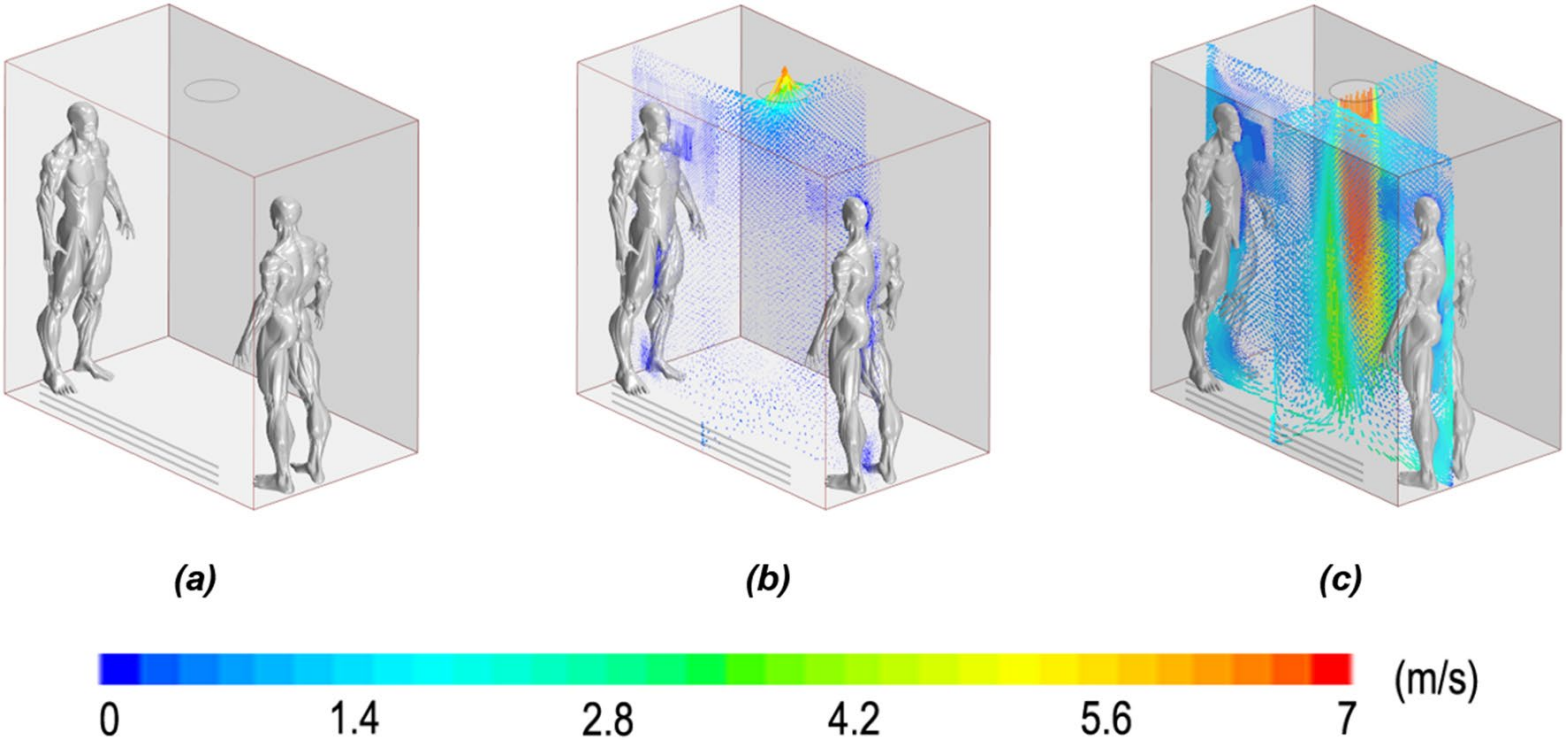
Velocity streamlines for the following cases: (**a**) no fan, (**b**) upwards-blowing fan and (**c**) downwards blowing fan.

**Figure 6:**
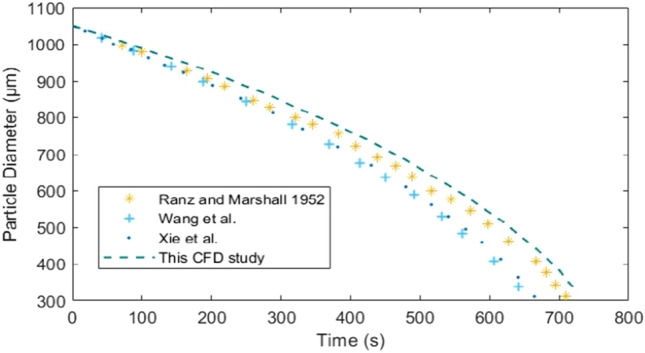
Comparison of the data collected with the designed configuration and other investigations, such as the experimental results from Ranz and Marshall^72^ and the numerical results from Wang et al.^67^ and Xie et al.^39^.

First, a steady simulation was created, introducing only airflow created with the ventilation into the elevator until the data converged. This continuum phase was modelled as Eulerian. The ambient conditions inside the lift were configured with the unique flow velocities of the configured fan, air supply racks and mouth inlets. For the case where the fan was used to exhaust the air inside, the device was set up as a pressure outlet with a value of − 35 pascals (Pa), creating a maximum velocity outlet of 6.5 m/s in the zone attached to the fan. In contrast, for the case of a downwards-blowing fan, the fan was configured as a velocity inlet with the same velocity of 6.5 m/s. For both cases, the racks were set up as stagnation inlets. The climatic conditions represent a clinical atmosphere with a constant temperature of 25 °C and a relative humidity of 50%. To maintain these conditions, the air introduced through the fan or racks had the same composition as the initial conditions (99.002% air; 0.998% H_2_O). Figure 6 shows the convergence results for three different cases: without a fan, with an upwards blowing fan and with a downwards blowing fan. The Reynolds-averaged Navier–Stokes (RANS) mathematical model was used for the entire computational domain, with the *k-ε* turbulence model, described by Alfonsi in^65^ (Fig. 5).

Second, once the airflow inside the elevator was in continuous motion, the simulation became implicit unsteady 2nd order. Therefore, the respiration and aerosols generated were modelled as the Lagrangian phase. The simulation is solved as a two-phase flow situation, introducing the two-way coupling module. Droplets, represented by particle clusters (parcels), are injected into the domain via surface injectors located at the emitter mouth. The samples were configured as pure water without any residue inside; thus, they directly disappeared from the domain when they were absolutely evaporated or when they touched any surface. The total injected mass flow was 6.7 mg according to the experiments carried out by Zhu et al.^66^. The injection occurred in 0.26 s. The diameter was selected based on the Rosin–Rammler distribution, as shown in Eq. (8). The selected diameter range was from 10 to 300 µm (µm), with a mean diameter of 80 µm.

8 $$D=\frac{n}{\overline{{d }_{g}}}{\left(\frac{{d}_{g}}{\overline{{d }_{g}}}\right)}^{n}{e}^{{-\left(\frac{d}{{d}_{g}}\right)}^{n}} ; n=8 , \overline{{d }_{g}}=80\mathrm{ \mu m}$$

where $D$ is the accumulated fraction of particles (μm) in the range of $d$ (10 to 300 µm). $n$ is the shape parameter, ${d}_{g}$ is the consider diameter (μm) and $\overline{{d }_{g}}$ is the mean diameter (μm).

The Taylor analogy breakup (TAB) rupture model was implemented to provide a solution to particle distortion and breakup. In addition, the turbulent particle dispersion with the exact eddy interaction time is considered. A quasi-stable model was implemented to address the phenomenon of evaporation. The droplets lose mass ${\dot{m}}_{p}$ (g/s) according to the following formula, Busco et al.^28^ and Ugarte-Anero et al.^15^

9 $${\dot{m}}_{p}={{\text{g}}}^{*}\times {A}_{s}{\text{ln}}\left(1+B\right)$$

where ${{\text{g}}}^{*}$ is the mass transfer conductance (g/m^2^s) and ${A}_{s}$ is the droplet surface area (m^2^). $B$ is the spacing transfer number.

Droplet motion was calculated using the Newton’s second law. The drag force (N), presented in Eq. (9), is calculated by the applied forces on a droplet as a function of its relative velocity inside the Eulerian phase. To obtain the drag force, the Schiller–Nauman correlation was employed (9).

10 $${F}_{D}=1/2{C}_{D}\uprho {A}_{P}{V}_{rel}^{2}$$

where ${C}_{D}$ is the drag coefficient, $\uprho$ is the density of air (kg/m^3^), ${A}_{P}$ is the projected area of the droplet (m^2^) and ${V}_{rel}$ is the relative velocity (m/s). The expression for calculating the drag coefficient is as follows:

11 $${{\text{C}}}_{{\text{d}}}\left\{\begin{array}{ll}\frac{24}{{\text{Re}}}, & \quad Re\le 1\\ \frac{24}{{\text{Re}}}\left(1+0.15{{\text{Re}}}^{0.687}\right), & \quad 1 < Re\le 1000\\ 0.44, & \quad Re>1000\end{array}\right.$$

where Re is the Reynolds number.

In addition, the gravitational force was also considered. Although the drag force is preeminent at the first moment from the start of the cough, when the cough is stopped, the buoyancy, gravity, the pressure gradient force and streams affect the droplet path.

As a droplet dispersion study, this was a classical unsteady problem. To solve this problem, the Courant number ($Co$) needed to be define and is shown in Eq. (10); this dimensionless number relates how many cells traverse a parcel or a droplet at each time step, $\Delta t$ (s), for a known velocity, $v$ (m/s). $\Delta x$ (m) represented the length interval.

12 $$Co=\frac{v\cdot \Delta t}{\Delta x}$$

To obtain an accurate solution, the Co needs to be equal or below to 1. The smallest cell (attached to the mouth cell) had a thickness of $\Delta x=5$ mm and the maximum obtained velocities were 4.4 m/s during breathing and 17.7 m/s during coughing; thus, Eq. (10) was used to determine that the selected values at $\Delta t=0.001$ s for the breathing period and at $\Delta t=2.3\cdot {10}^{-4}$ s for the coughing phase (from second 5 to second 5.5) were valid to produce an accurate result.

The commercial CFD code STAR-CCM + v.14.02 (Siemens, London, UK) were used to define and solve the numerical problems that were defined to study CO_2_ accumulation and droplet dispersion. A personal server-clustered parallel computer with an Intel Xeon © E5-2609 v2 CPU @ 2.5 GHz (16 cores) and 45 GB of RAM was used to run all simulations.

### Validation

A mathematical model based on computational fluid techniques needed to be validated with the experimental results, and similar results were obtained. Therefore, an experimental study was selected. For this purpose, the phenomenon of evaporation was observed by studying the reduction in the diameter of the droplet. The predicted results from Rand and Marshall (1952) were consistent with the obtained results. This investigation investigated the evaporation of stationary water droplets in a dry environment (T_∞_ = 25 °C, RH = 0%). The initial temperature of the droplet is 9 °C. Figure 6 shows a comparison of the experimental data, the data from the model of Wang et al.^67^, the data from Xie et al.^39^, and the data obtained with the designed configuration.

## Results

### CO_2_ concentration

**Figure 7:**
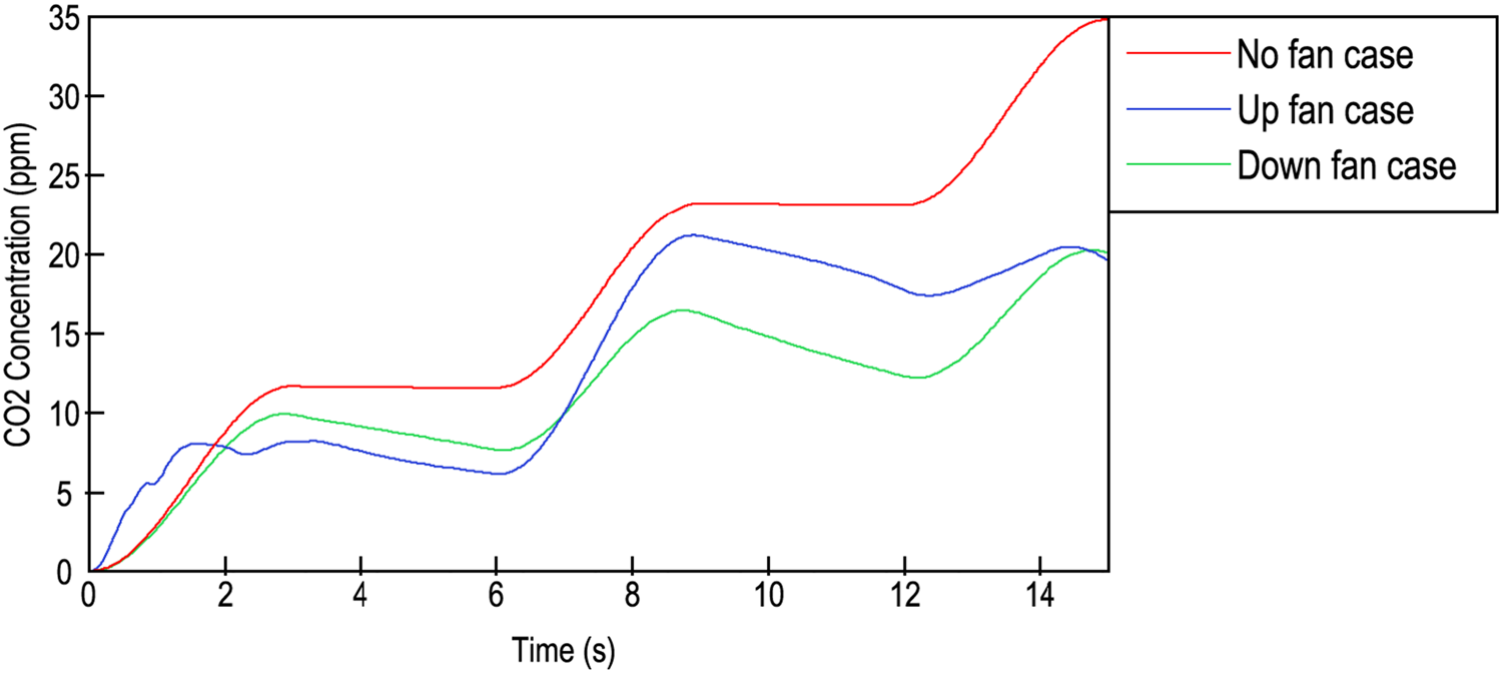
CO_2_ concentration inside the lift. Red line: no fan; blue line: upwards-blowing case; green line: downwards-blowing case.

Figure 7 shows the increase in the CO_2_ concentration measured in mass parts per million (ppm) during the 15 s journey. The unique CO_2_ source was the person coughing, who expelled CO_2_ with other gases in a sinusoidal breathing shape. In the NF case, an increase in CO_2_ had a pure sinusoidal accumulative shape, reaching a maximum value of 35 ppm in 15 s; this occurred at the instant when the simulation finished. For cases where ventilation existed, the concentration was lower than that in the case of NF for the entire time, with the exception of the first second of the UF case, where the concentration was unexpectedly higher than that in the case of no ventilation.

The incidence of ventilation was slightly different among the two groups. Since the CO_2_ total expulsion was triggered in three cycles in the first two cycles, the UF case had the highest increasing ratio. In contrast, the decrease in the CO_2_ concentration observed in the moments where breathing process was in aspiration phase were similar for both ventilated cases. At the 13th second, the CO_2_ exhausting stream was established for UF case. This was likely due to the minimal increase in the last exhaust cycle for the UF case compared to that for the DF case. In the last second, both ventilation systems reached the same value of 20.2 ppm. Interestingly, the no fan and downwards-blowing fan cases were totally cyclic and harmonious, while the upward-blowing fan case had few irregularities, and the cycles were not homogenous.

For more information, see the supplementary material, where CO_2_ transport was modelled and studied.

### CO_2_-based infection probability

Equation (11) describes a validated solution presented in Wang et al.^68^ and Foster and Kinzel^69^ to calculate the probability of infection in a closed space. This equation depends on the emitted quanta, which is the number of contagious aerosols emitted per hour, with one quantum being the necessary amount to be infected. Additionally, the emitted quanta depend on the CO_2_ concentration and exposure time. For this study, a 20-quantum per hour emission rate was estimated based on the proposed values of^68^ and^69^, who implemented 14 and 100 quantum per hour, respectively.

13 $$P=1-{e}^{(-q\cdot Y\cdot t)}$$

where ${\text{P}}$ is the probability of being infected, ${\text{q}}$ is the quanta emission rate (h^−1^), ${\text{Y}}$ is the CO_2_ concentration over time (ppm) and ${\text{t}}$ is the exposure time (h).

**Figure 8:**
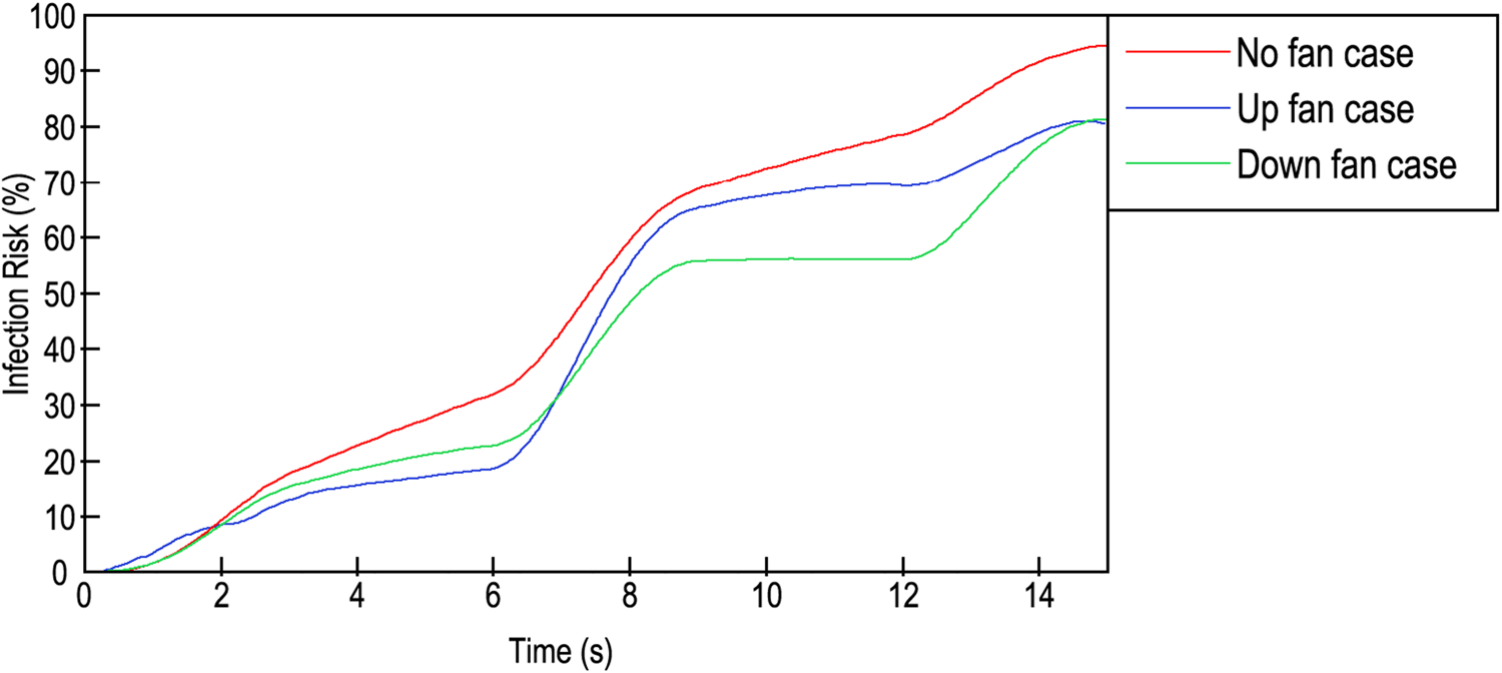
CO_2_-based infection probability. Red line: no fan; blue line: upwards-blowing case; green line: downwards-blowing case.

Figure 8 shows the calculated probability of contagion for the proposed scenarios for the entire time period based on Eq. (11). Along 15 s, the no fan case was always the riskiest scenario; the CO_2_ concentration increased, reaching a maximum probability of COVID-19 infection of 94.5%. The values for the upwards-blowing fan case and downwards-blowing fan case were similar until the 7th second; here, the upwards blowing fan case increased slightly more than downwards blowing fan case, at around 12% until 12.5th second. Beyond this moment, both probabilities converged to similar values of 80.6% for the upwards-blowing fan case and 81.2% for the downwards-blowing fan case in the last second. For three scenarios, the increase in risk was linked to CO_2_ exhalation in higher amounts rather than to the exposure time. The differences in the gradients between the exhalation moments and inhalation moments were apparent.

### Droplet quantity fraction

**Figure 9:**
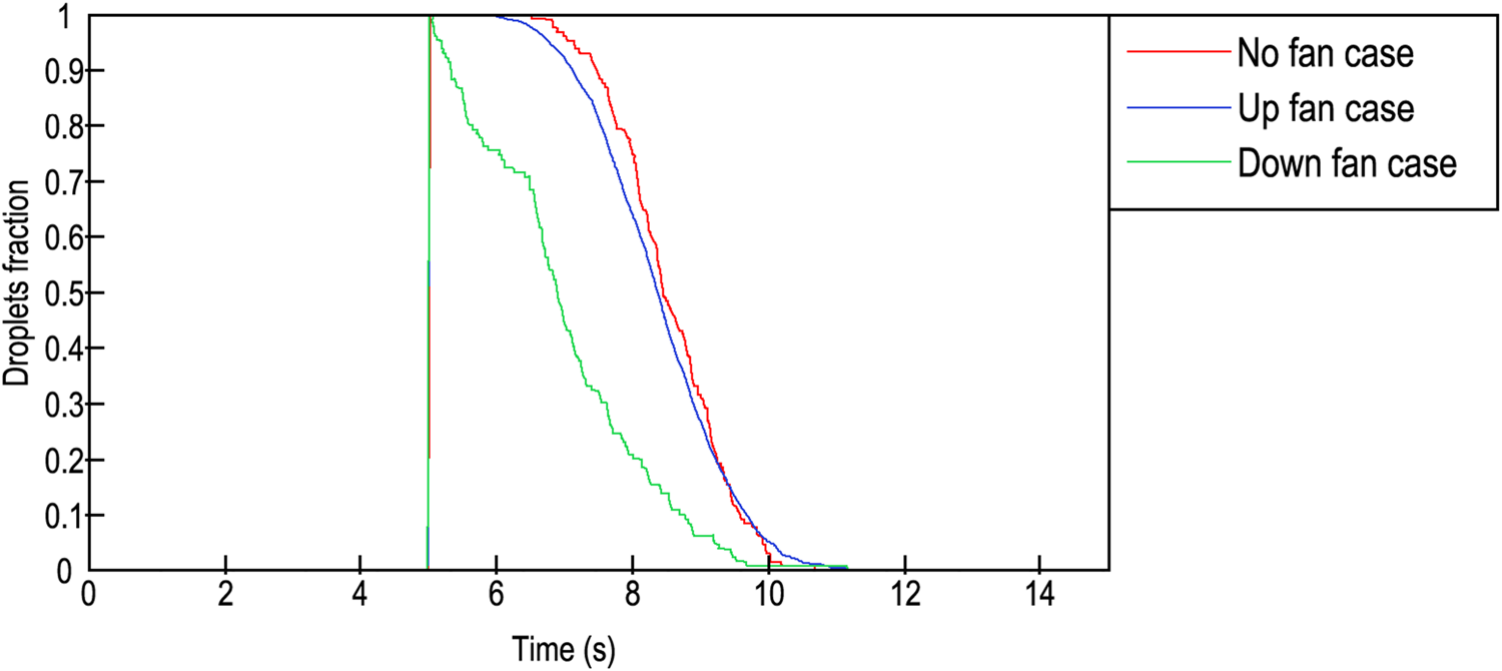
Droplets fraction inside the lift. Red line: no fan; blue line: upwards-blowing case; green line: downwards-blowing case.

Figure 9 shows that the droplets missing from the domain are expressed as a fraction of 1. In three cases, droplets appeared suddenly in the 5th second. The graph indicates that the DF case largely differed from the UF case and the NF case. According to the image, in the downwards-blowing fan case, 30% of the droplets evaporated or were quickly expelled in one and a half seconds, respectively, and the removal was noticeable from the first moment. Instead, the upwards-blowing case needed two seconds to eliminate only 10% of the droplets; moreover, no fan case lasted 2.4 s to evaporate the same quantity of droplets. In the UF and NF cases, the removal started 1 s and 1.5 s, respectively, after the cough began.

Ninety percent of the droplets were removed within 3.5 s from the beginning of the cough in the DF case. This time was delayed to 4.5 s for both the NF and UF cases. On the other hand, the total quantity of evaporated or expelled droplets occurred approximately at the same time for the three cases, in 5.6 s for the NF case and 6 s for the two cases with ventilation.

**Figure 10:**
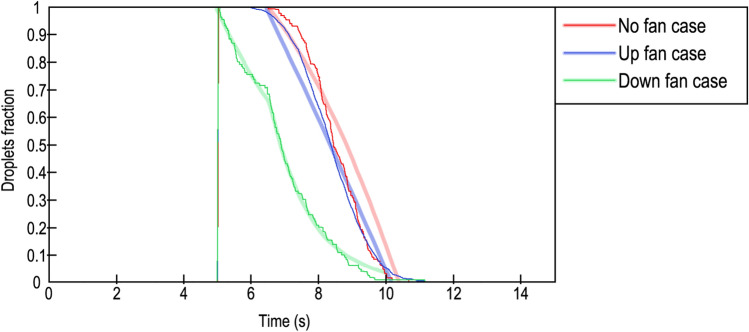
Comparison of droplet fractions between CFD results (solid lines) and predicted results from Table 5 (blurred lines). Red line: No fan case; blue line: upwards blowing case; green line: downwards blowing case.

Finally, some differences between elimination ratios were detected. In the DF case, droplets were removed in two negative exponential frames, while in the UF and NF cases, droplets were removed in a parabolic form. Table 5 provides the fitting equations and main parameters such as the confidence bound, R^2^ and the root mean squared error (RMSE). Equations were proposed presuming that the cough started at second 0. The plotted results can be found in Fig. 10.

**Table 5:** Fitting equations and parameters.

Curve	Equation	a	b	c	Confidence bound (%)	${R}^{2}$	RMSE
No fan 1	$f\left(x\right)=a\cdot {e}^{bx}$	0.975	− 0.261	–	95	0.9528	0.02
No fan 2	$f\left(x\right)=a\cdot {e}^{bx}$	2.394	− 0.837	–	95	0.9954	0.01
Up fan	$f\left(x\right)=a{x}^{2}+bx+c$	− 0.004	− 0.244	1.37	95	0.9555	0.07
Down fan	$f\left(x\right)=a{x}^{2}+bx+c$	− 0.037	− 0.064	1.205	95	0.9607	0.06

### Droplet dispersion

In this subsection, the propagation of droplets for different ventilation systems was visually compared. The defined images were chosen for three phases, where the total exhalation, a well-expanded dispersion and, finally, the last remaining droplets were well defined. According to this proposition, the 6th second, 8th second and 10th second were projected (the cough started in second 5). The same time-configuration was adopted for all ventilation strategies, making it possible to compare them and evaluate which was the optimal set up in terms of dispersion. Frontal and perspective images are presented for a better comprehension of the volume dispersion.

**Figure 11:**
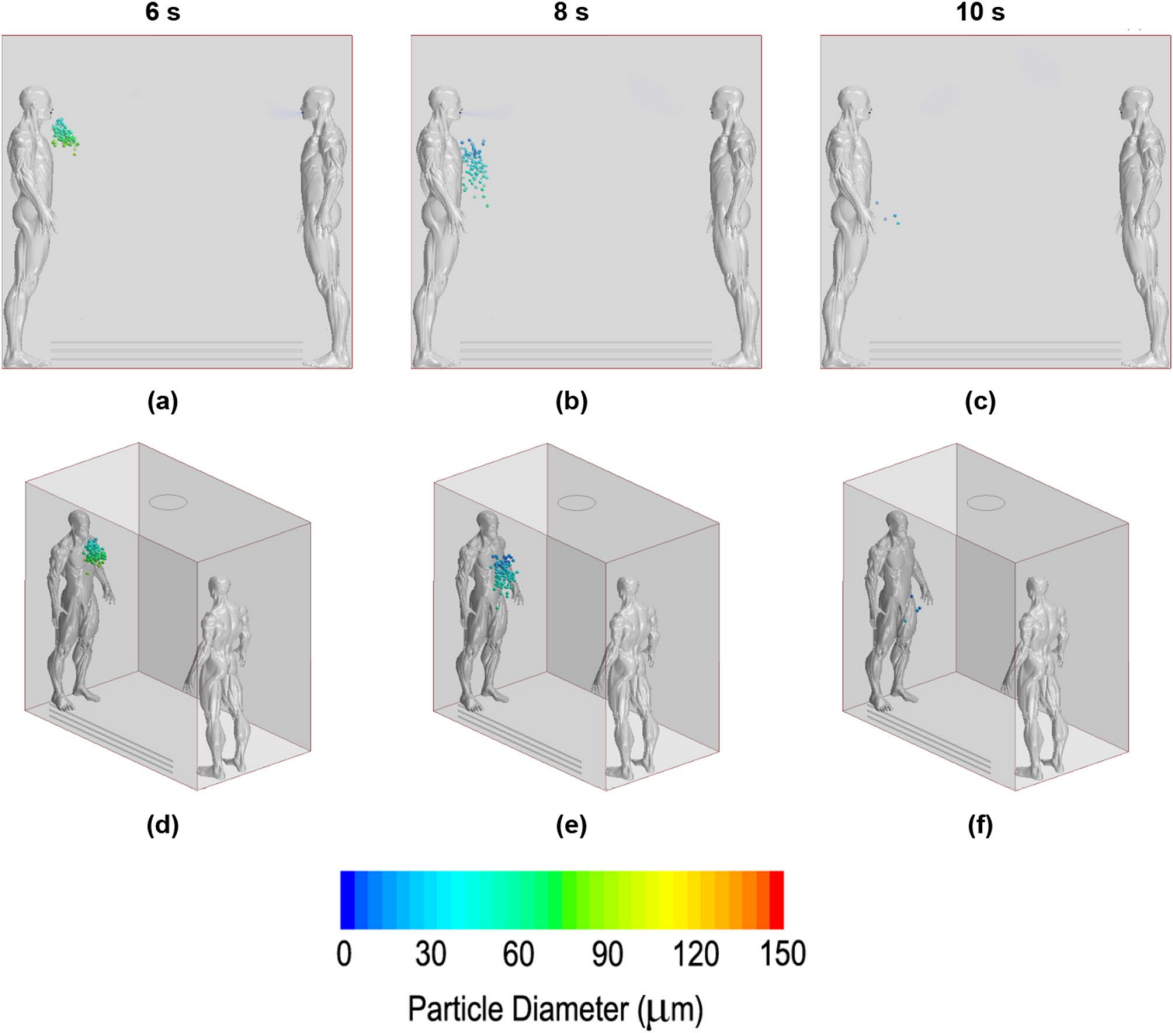
Droplet dispersion inside the lift for the no fan case. (**a**) and (**d**) in 6th second; (**b**) and (**e**) in 8th second; (**c**) and (**f**) in 10th second. First row of images for frontal point view. Second image row for perspective point view.

Figure 11 shows the results for the no fan case. The entire droplet cloud was observed outside the mouth at a few centimetres below the mouth in the 6th second. Two seconds later, the cloud continued to fall due to gravity and was slowed by drag and buoyancy. During this time, the particles decreased in size and number due to evaporation. In the last second, very few particles remained inside the domain according to the height of the subject’s waist. For this no-fan case, the unique horizontal displacement was due to the initial cough jet. Once the drag balanced the displacement in this axis, no force was able to move any particle.

**Figure 12:**
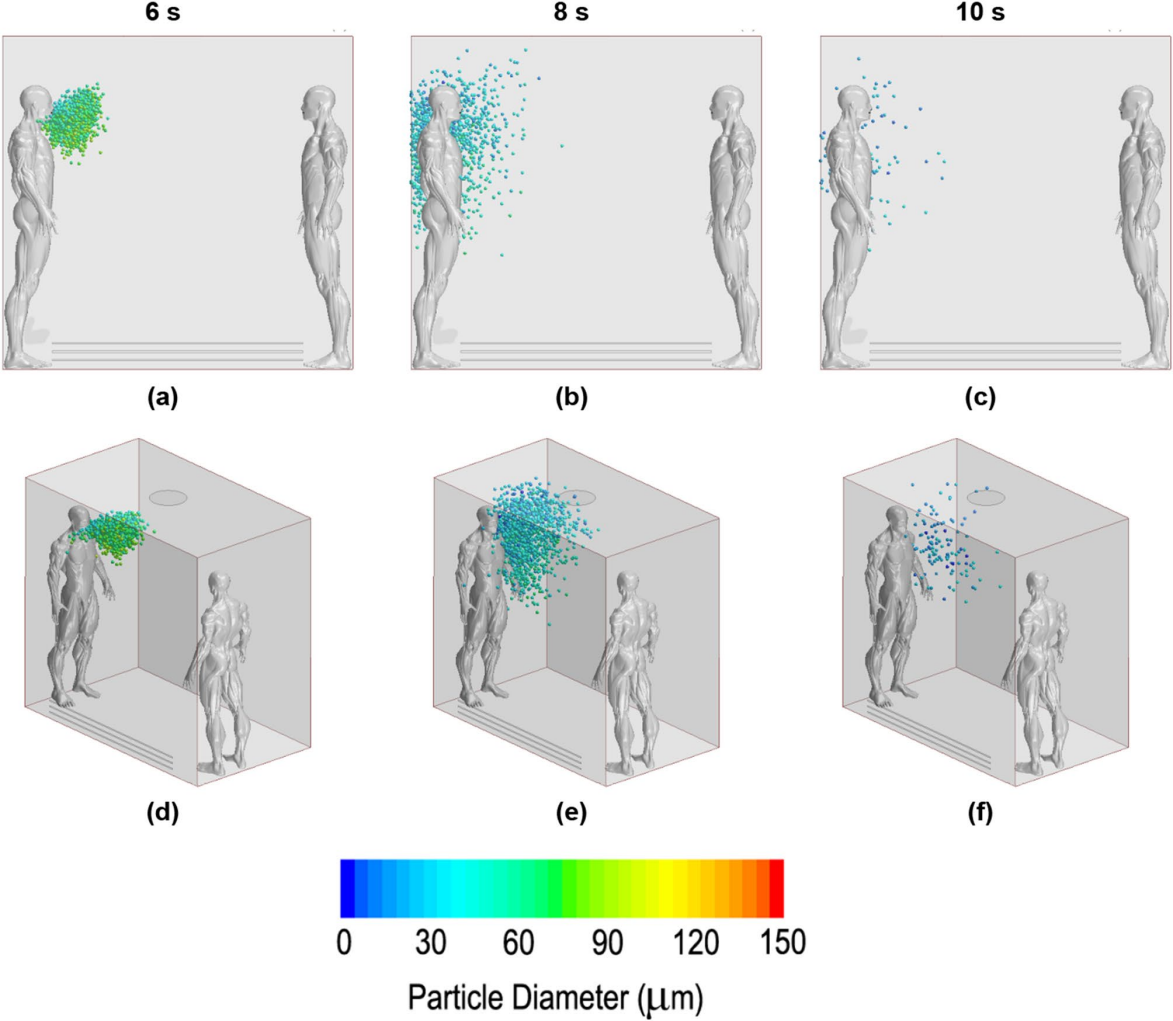
Droplet dispersion inside the lift for the upwards-blowing fan case. (**a**) and (**d**) in 6th second; (**b**) and (**e**) in 8th second; (**c**) and (**f**) in 10th second. First row of images for frontal point view. Second image row for perspective point view.

Figure 12 shows the results for the upwards-blowing fan case. The droplets travelled forward the first moment from the beginning of the cough. Immediately, they were attracted by the exhausting stream but inside a recirculation behaviour that positioned them at the back of the emitter and at his left side. In this case, several particles maintained a high position over the head of the infected person. No droplet reached the second occupant due to the mentioned recirculation. Particles near the exhausting fan disappeared before being expelled due to evaporation.

**Figure 13:**
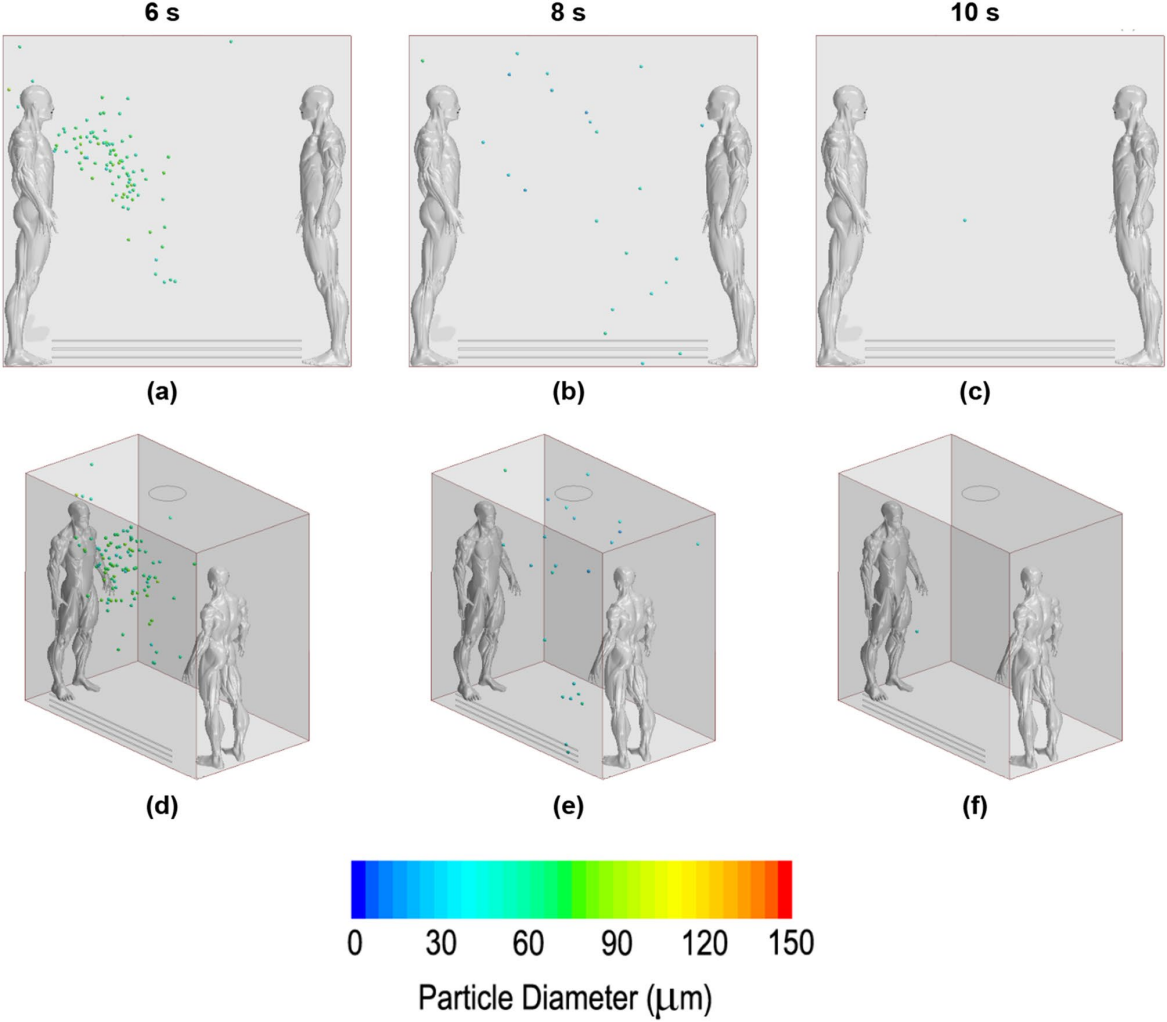
Droplet dispersion inside the lift for the downwards-blowing fan case. (**a**) and (**d**) in 6th second; (**b**) and (**e**) in 8th second; (**c**) and (**f**) in 10th second. First row of images for frontal point view. Second image row for perspective point view.

Figure 13 shows the downwards-blowing fan performance. The pictures explain the induced movements on the droplets. From the beginning, the particles moved downwards rapidly, quickly impacting on the bottom surface and becoming attached there. Few particles entered recirculation and were capable of going up after passing through the stream without being stuck on the floor or evaporated, reaching positions near the second occupant. For this case, the dispersion could be considered total but at a low level; most of the particles evaporated quickly, but few particles reached positions far away from the source in all Cartesian axes.

## Discussion and study limitations

The presented CFD study is applicable to a possible but poorly likely scenario in which two subjects face each other and share a clinical lift where one of them coughs. Additionally, it is noticeable that only the person coughing exhaled CO_2_. The purpose of this hypothesis was to investigate the airborne concentration coming from the infected person. Carbon dioxide concentrations were measured in mass ppm, and the initial concentration was zero.

The simplification of generating a circular hole with negative pressure (downwards-blowing fan case) or velocity inlet (upwards-blowing fan case) could alter the real results. The simulated case does not generate the typical helical streams of a fan. Nevertheless, according to Shinohara et al.^50^, this approximation is valid for accurately calculating the CO_2_ concentration but not for calculating the gas mixing homogeneity inside the domain due to the lack of turbulence. The supply of air has an important effect on interactions with respiratory flows. For the DF case, the effect of the supply air was immediate since the flow directly affected the CO_2_ emission and droplet dispersion. However, for the UF case, the renewed air did not quickly affect either the gas or droplet emissions. For this case, the supply air entered from below and crossed a large horizontal area until it reached the top; thus, the effect was not centred if not near the wall positioned in the face of the racks.

According to the obtained CO_2_ concentration results, the maximum obtained value was 35 ppm in the no fan case. In contrast, downwards-blowing fan ventilation appears to be the most suitable approach. This result can be debateable because the exhaust area is less than that in the upwards-blowing fan case (0.045 m^2^ < 0.07 m^2^). However, this result was reported by Rodriguez et al.^11^, whose recommendation was to set up the inlet supply air at the top and the outlet at the bottom. This result could differ from the 15th second onwards, when the two proposed ventilation setups coincided at 20.2 ppm, and immediately after, the upwards-blowing fan values were lower than those of the downwards-blowing fan case. Considering the results, the study could be expanded to longer time frames. The results show a unique value for the entire computational domain volume. In future works, the authors recommend discretizing the elevator domain into different height-volume controls to identify the riskiest zones.

The ability of droplets to be removed was evaluated fractionally. Droplets appeared immediately (in 0.23 s). The evaporation, deposition or expulsion data affirmed that the downwards-blowing fan had the best performance. In contrast, the no fan case took the longest to start eliminating droplets. In these terms, the upwards-blowing fan case had slightly greater effectiveness than the no ventilation setup. Notably, 100% of the droplets nearly disappeared at the same time, between 5.6 and 6.1 s after the start of coughing, which was consistent with the results of Yang et al.^10^. In addition, aerosols are composed of different components that form the fluid of saliva, according to Carpenter et al.^70^. This fluid is formed by water and nonvolatile solids^71^. The particles generated by the subjects of this study were simulated as pure water, as in the CFD study of Dbouk et al.^59^. The software used has the limitation of not being able to simulate the nonvolatile solid components of the fluid. Therefore, the particles created for this study evaporated completely when they lost all the liquid and were removed from the domain. This is not entirely true since nonvolatile components that can contain viruses would still exist in the environment. Thus, it is necessary to know that the results shown in this research do not reveal aerosols with volatile components but rather reveal aerosols with liquids.

Four linear regressions were proposed (2 for the downwards-blowing fan case, 1 for the upwards-blowing fan case and 1 for the no fan case data). The variability of ${R}^{2}$ for the proposed equations was sufficiently accurate (between 0.9528 and 0.9954). These values are not representative of the different cases presented here. Therefore, it would be recommended and interesting to perform tests with different variables, such as different relative humidities, droplet emission sources, ventilation setups, and ventilation ratios. This information could be a useful tool or database for studying and predicting better ventilation systems in small closed spaces.

In relation to the ventilation performance under droplet dispersion, it was remarkable that the safest scenario could be the strategy without mechanical ventilation. The dispersion in the no fan case was the smallest in comparison with that in the other two cases. This was because the unique propagation forces were due to the initial cough jet. When this puff finished, the droplets fell down vertically by gravitational force with the unique opposition of the air drag force. In this case, the healthy passenger was safe from direct contagion. The upwards-blowing fan case had greater dispersion, but the droplets were concentrated above all in the emitter region. The recirculation caused by the fan maintained particles near the origin zone, making the area of the healthy subject relatively safe in terms of direct infection. Finally, the downwards blowing fan quickly impacted most of the droplets on the floor, removing them from the domain. The issue in this scenario was that a few particles could avoid collision, recirculate inside the lift and even increase the healthy passenger respiration volume, facilitating direct infection.

## Conclusion

In the present study, 3 CFD cases were compared. A lift under a typical clinical atmosphere and standard size, with two standing subjects, was modelled. Both breathed in a sinusoidal shape, but only one (the infected person) exhaled CO_2_. After 5 s, the infected person coughed, and the droplets were released into the air. Three scenarios were compared: without ventilation, with an upwards-blowing fan, and with a downwards-blowing fan. Finally, the mass CO_2_ concentration, the amount of cough particles removed and droplets dispersion were compared and analysed for the different ventilation cases. The results showed the following:

- The no ventilation case was less effective at exhausting CO_2_, reaching 35 ppm in 15 s. The downwards-blowing fan case expelled the most gas. Similar conclusions were drawn from the risk probability study. The no ventilation case was associated with a higher contagion rate; moreover, it was difficult to conclude that downwards-blowing ventilation case was safer than the upwards-blowing fan case.
- With respect to droplet deletion, the best case was a downwards-blowing fan system, which removed all droplets more quickly. However, the other two cases exhibited similar trends with the upwards-blowing fan configuration being slightly more effective.
- Linear regressions were developed to adjust the obtained results to simple mathematical models, and a minimum acceptable precision of ${R}^{2}$= 0.9528 was obtained.
- In terms of liquid droplet dispersion, the no ventilation scenario appeared to be the safest. All droplets fell down and evaporated before reaching the floor.

### Supplementary Information

Supplementary Information.

## Data Availability

The data presented in this study are available upon request from the corresponding author.

## List of symbols

A_p_: Projected area of the droplet [m^2^]
A_s_: Droplet surface area [m^2^]
$B$: Spacing transfer number [–]
$${C}_{D}$$: Drag coefficient [–]
$$Co$$: Courant number [–]
$D$: Accumulated fraction of particles [μm]
d: Diameter range [μm]
${d}_{g}$: Consider diameter [μm]
$\overline{{d }_{g}}$: Mean diameter [μm]
$${F}_{D}$$: Drag force [N]
${{\text{g}}}^{*}$: Mass transfer conductance [g/m^2^s]
${\dot{m}}_{p}$: Mass of droplets [g/s]
$$n$$: Shape parameter [–]
$p$: Order of converge [–]
${\text{P}}$: Probability of risk of infection [–]
${\text{q}}$: Quanta emission rate [h^−1^]
$r$: Mesh refinement ratio [–]
Re: Reynolds number [–]
$${\text{t}}$$: Exposure time [h]
T_∞_: Environment temperature [°C]
$$v$$: Velocity [m/s]
$${V}_{ax}$$: Axial velocity [m/s]
$${V}_{rel}$$: Relative velocity [m/s]
${\text{Y}}$: CO_2_ concentration over time [ppm]
$$\Delta t$$: Time step [t]
$$\Delta x$$: Length interval [m]
$$\in $$: Relative error [–]
$$\uprho $$: Density [kg/m^3^]

## Quantity

Length: Meter [m]
Area (Length^2^): Square meter [m^2^]
Volume (Length^3^): Cubic meter [m^3^]
Mass: Gram [g]
Time: Second [s]
Temperature: Celsius [°C]
Velocity (Length/Time): Meter/second [m/s]
Volume: Parts per million [ppm]
Volume/Time: Flow [Litre/minute]
Pressure: Pascal [Pa]
Force: Kilogram × meter/square second [N]

## Abbreviations

CFD: Computational fluid dynamics
CO_2_: Carbon dioxide
DF: Downwards blowing ventilation
EN: European standards
GCI: Grid converge index
NF: Without ventilation
RANS: Reynolds averaged Navier–Stokes
RH: Relative humidity
RMSE: Root mean squared error
TAB: Taylor analogy breakup
UF: Upwards blowing ventilation
VC: Volume control
RANS: Reynolds averaged Navier Stokes
