# Supplementary material for: Numerical performance of CO2 accumulation and droplet dispersion from a cough inside a hospital lift under different ventilation strategies
Source: Sci Rep. 2024 Mar 21;14:6843. doi: 10.1038/s41598-024-57425-z (PMC10957917; doi:10.1038/s41598-024-57425-z)
Supplement: Supplementary file 1 — Supplementary Information. [file 41598_2024_57425_MOESM1_ESM.docx]

Numerical performance of CO_2_ accumulation and droplet dispersion from a cough inside a hospital lift under different ventilation strategies

1. Supplementary material

This section shows the transport of CO_2_ inside the lift and how the ventilation affects the movement of gas. As explained in the main text, CO_2_ gas can be defined as aerosol tracer. With the purpose of analyse the movement of nuclei in the elevator, the exhaled CO_2_ by the infected person was modelled and defined as derived part. Figure A1 shows the obtained results for the moments 2 seconds, 5 seconds and 10 seconds for the three carried out cases (no fan case, upward−blowing fan and downward−blowing fan respectively).

| 2 s | 5 s | 10 s |
| --- | --- | --- |
| 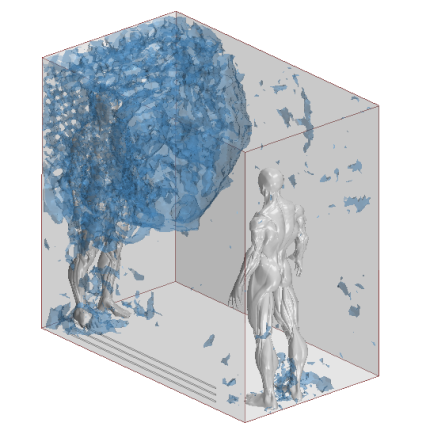 | 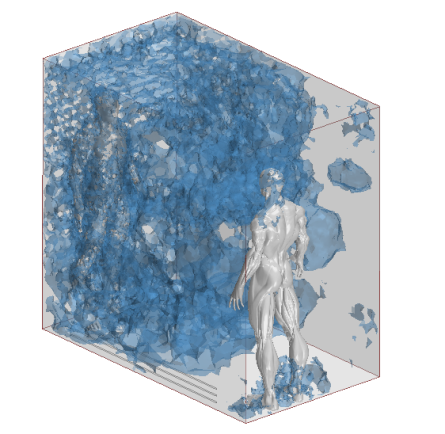 | 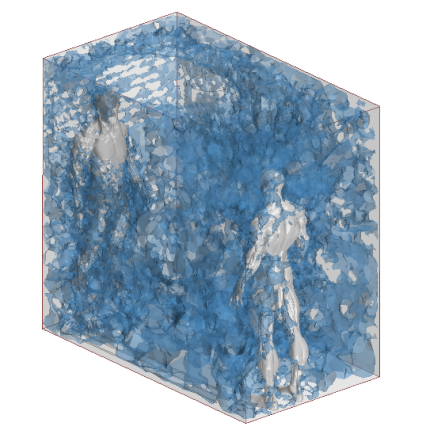 |
| (a) | (b) | (c) |
| 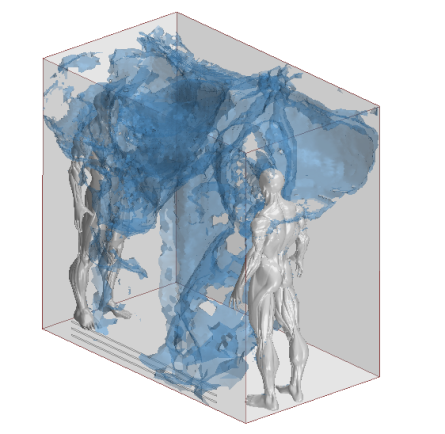 | 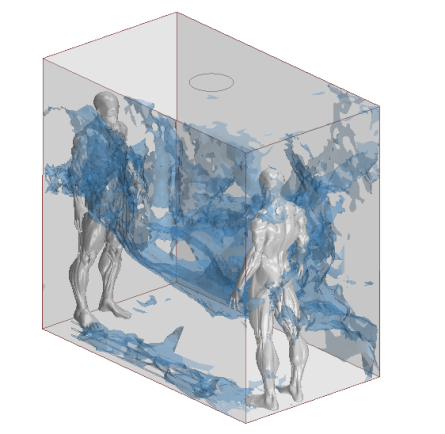 | 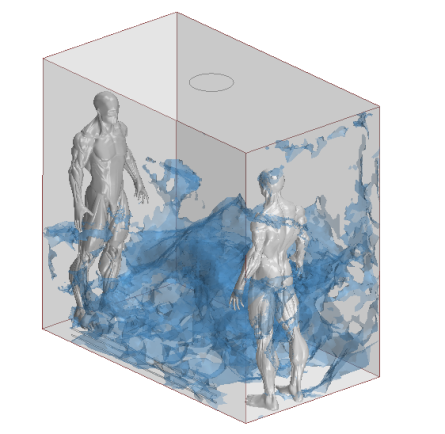 |
| (d) | (e) | (f) |
| 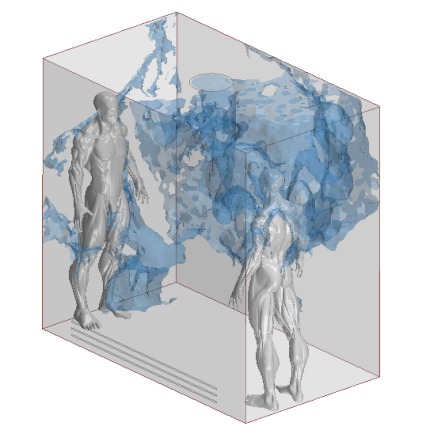 | 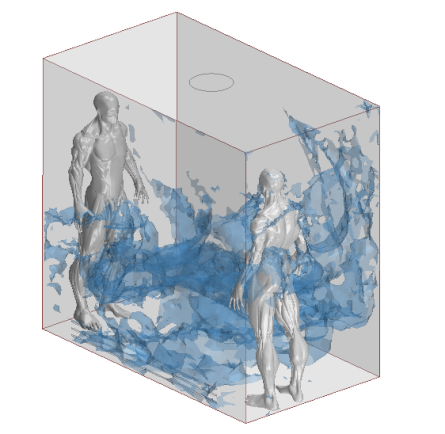 | 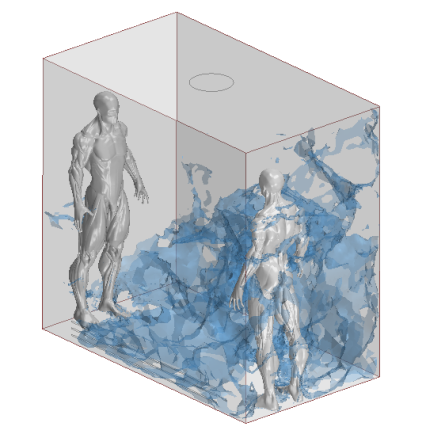 |
| (g) | (h) | (i) |

**Figure A1.** CO_2_ transport inside the lift. (**a**), (**b**) and (**c**) pictures shows the results for no fan case in seconds 2, 5 and 10 respectively; (**d**), (**e**) and (**f**) images correspond to the results for upward−blowing fan case and in the same times and (**g**), (**h**) and (**i**) images correspond to the result of downward−blowing fan case for the same moments.

CO_2_ transport images show how the carbon dioxide, and therefore aerosol nuclei, got dispersed inside the lift for the three different ventilation strategies. There, it can be seen that no fan case was the one which obtained the greatest dispersion, filling the entire cabin and reaching the healthy occupant in 5 seconds. At the beginning of the simulation, a semi−spherical shaped CO_2_ cloud covered the emitter passenger, and afterwards, due to the lack of ventilation, the cloud grew up reaching every zone of the lift.

For cases where ventilation existed, CO_2_ dispersion was similar in transport as in concentration, in concordance with the obtained results in 3.1 subsection (CO_2_ concentration). In the second 2, the gas was dispersed by the height of the emitter head; in higher height for upward−blowing fan case. In the second 5, results showed that due to streams recirculation and possibly to density difference with the ambient air (CO_2_ is denser than air), this gas tended to occupy the low region. In the last considered moments, CO_2_ was positioned overall in the zone of the healthy occupant, away from the emitter and reaching heights near to shoulders for both ventilated cases.

In relation to CO_2_ transport, it could be concluded that any ventilation and air renewal gave safer environment than the case without air circulation, for concentrations and risky zones.
